# Counting the Cost: The Hidden Financial Realities of Neuromuscular Disease Through Patient and Family Perspectives

**DOI:** 10.1111/hex.70529

**Published:** 2025-12-17

**Authors:** Homira Osman, Zainab Adamji, Stacey Lintern, Ian C. Smith, Alyssa Grant, Lola E.R. Lessard, Hanns Lochmuller, Hugh McMillan, Kathryn Selby, Gerald Pfeffer, Lawrence Korngut, Cynthia Gagnon, Kednapa Thavorn, Jodi Warman‐Chardon

**Affiliations:** ^1^ Muscular Dystrophy Canada Toronto Ontario Canada; ^2^ The Ottawa Hospital Research Institute Ottawa Ontario Canada; ^3^ Faculty of Medicine University of Ottawa Ottawa Ontario Canada; ^4^ Department of Medicine (Neurology) The Ottawa Hospital/University of Ottawa Ottawa Ontario Canada; ^5^ Children's Hospital of Eastern Ontario Ottawa Ontario Canada; ^6^ Department of Clinical Neurosciences Hotchkiss Brain Institute Calgary Alberta Canada; ^7^ Department of Medical Genetics Alberta Child Health Research Institute Calgary Alberta Canada; ^8^ University of Sherbrooke Sherbrooke Quebec Canada; ^9^ British Columbia Children's Hospital Vancouver British Columbia Canada; ^10^ School of Epidemiology and Public Health University of Ottawa Ottawa Ontario Canada

**Keywords:** financial impact, neuromuscular disease, patient lived experience, quality of life, rare disease, socio‐economic burden

## Abstract

**Introduction:**

Neuromuscular diseases (NMDs) impose multifaceted challenges on individuals and their families, often resulting in significant medical and non‐medical expenses. While cost‐of‐illness (COI) studies provide valuable quantitative data, few explore the lived experience of financial strain. This study aims to identify the complex, often hidden, financial impacts experienced by individuals with NMDs and their families.

**Methods:**

We conducted a qualitative study involving four virtual semi‐structured focus groups, with 58 participants (76% patients and 24% caregivers). Participants were recruited from Muscular Dystrophy Canada's database and had previously completed the national BIND COI survey. Participants shared firsthand accounts of direct non‐medical costs, psychosocial burdens and opportunity costs, highlighting hidden expenses, substantial out‐of‐pocket costs, and the broader financial and emotional toll on families. Thematic analysis of the transcripts of the discussions was performed using an inductive approach, guided by a rare‐disease‐specific socio‐economic burden framework.

**Results:**

Four key themes emerged: informational costs (lack of awareness/support for navigating financial resources), time‐related costs (time spent advocating for supports), opportunity costs (loss of income or career advancement), and costs to independence (emotional toll and out‐of‐pocket costs for assistive devices and home modifications).

**Conclusion:**

This study identified four interconnected categories of hidden costs for people with NMDs and their families: informational burdens, administrative and advocacy demands, employment‐related opportunity costs, and reduced independence tied to out‐of‐pocket spending on equipment and home modifications. These findings reveal how financial and emotional pressures accumulate beyond what traditional COI estimates capture. Greater attention to these costs is critical for fostering equitable and sustainable healthcare systems.

**Patient or Public Contribution:**

This study was designed and conducted in partnership with individuals living with NMDs and caregivers. Three trained patient and family research partners contributed to the development of the focus group guide and interpretation of results. All participants contributed their lived experiences to inform and validate key findings. Their input was central to the design, analysis and preparation of this manuscript, ensuring alignment with community priorities.

## 1 Introduction

Neuromuscular diseases (NMDs) are a heterogeneous group of rare, progressive disorders that impair skeletal muscle and peripheral nerve function. They often lead to severe physical disability, multisystem complications and reduced life expectancy. These conditions include hereditary and autoimmune progressive muscle and nerve diseases, and they typically require complex, lifelong management involving multidisciplinary care, respiratory and cardiac monitoring, rehabilitation, and assistive devices/technologies. Although advances in pharmacologic treatments, supportive care and medical devices have improved survival and quality of life, these improvements have introduced new financial pressures [1, 2, 3, 4]. Individuals living with NMDs and their families frequently face significant medical and non‐medical expenses, including out‐of‐pocket costs, lost income and caregiving responsibilities [2, 4, 5, 6].

In Canada, publicly funded healthcare is delivered through provincial and territorial health insurance plans [7]. Approximately 70% of healthcare expenditures, including physician services, diagnostic testing and hospital care, are covered through the public system. The remaining 30% includes services that are not universally insured, such as outpatient prescription drugs, rehabilitation therapies, complex assistive devices, dental and vision care, and many home‐ and community‐based supports [2]. These services are financed either through private insurance or directly by patients and caregivers. Recent estimates from the Canadian Institute for Health Information (CIHI) indicate that average per capita out‐of‐pocket spending is $1243 annually, while private insurance spending averages $993 [8]. Eligibility for publicly funded services varies substantially across provinces, particularly for assistive devices, home care, mobility equipment and rehabilitation therapies. Many supports require means testing, diagnostic confirmation or repeated reassessments, and reimbursement levels differ significantly between programmes. Federal supports such as the Disability Tax Credit and the Medical Expense Tax Credit can offset some costs but require complex applications and have restrictions on eligible expenses. As a result, even in a publicly funded healthcare system, people with chronic, progressive conditions such as NMDs experience substantial and variable cost exposure [9].

In this study, ‘hidden costs’ refer to the financial, administrative and psychosocial burdens that are not routinely captured in conventional economic evaluations or accounted for in healthcare system planning. We conceptualise hidden costs as the under‐recognised, non‐reimbursed demands that arise as individuals and families navigate the health, social, disability and education systems. These burdens extend beyond direct medical and indirect productivity‐related costs and include time investment, information gaps, administrative navigation, self‐advocacy and emotional strain. Drawing from literature on administrative burden, hidden costs encompass learning costs and compliance demands, such as acquiring disease‐related knowledge, interpreting eligibility rules, coordinating appointments, securing equipment and completing extensive paperwork [10, 11]. They also include information gaps and self‐advocacy work, where patients and caregivers must locate resources, negotiate access and challenge barriers within the healthcare, education and social support systems [12]. Finally, hidden costs involve psychosocial strain, including distress, uncertainty, decision fatigue and the cumulative emotional impact of ongoing care responsibilities [13, 14].

Most cost‐of‐illness (COI) studies in NMDs focus on direct medical costs, such as medication, hospital admissions and specialist consultations [15, 16]. However, individuals living with conditions like spinal muscular atrophy (SMA) and Duchenne muscular dystrophy (DMD) also face substantial direct non‐medical costs, which include home modifications, mobility and respiratory equipment, and travel and accommodation for treatment [2]. Beyond these, indirect costs represent a significant portion of the total socio‐economic burden. These include lost income from reduced employment or early retirement, absenteeism, decreased workforce participation among caregivers, and the costs associated with informal, unpaid caregiving. In several NMDs, including SMA, DMD and myasthenia gravis (MG), indirect costs have been estimated to account for 45%–80% of the overall disease burden [17, 18, 19]. Such costs often result from navigating fragmented systems, delays in accessing essential services, and the need to pay out‐of‐pocket for supports not covered by public or private insurance programmes.

While COI studies for various NMDs exist [2, 4, 5, 20, 21, 22], very few describe the socio‐economic impacts from the patient and family perspectives. In rare disease COI studies, nearly half (48%) rely on databases or registries to estimate costs [16]. However, these sources often fail to capture the full spectrum of direct and indirect costs, such as labour productivity losses and costs associated with informal caregiving or assistive device/equipment [16]. Cost estimates can also vary significantly depending on whether important costs such as home and vehicle renovations or allied healthcare professional (HCP) services are included [23]. Furthermore, the quality of data in administrative databases is limited by inconsistencies in data collection, diagnosis coding errors or systematic reporting biases [24, 25, 26].

Qualitative studies provide patient‐driven insights that reflect the diverse lived experiences shaped by factors such as the type and severity of the NMD, disease progression and the availability of resources. Understanding the full extent of unmeasured and indirect costs incurred by patients is essential for identifying critical gaps in care, informing cost‐effectiveness analyses and accurately capturing the comprehensive economic burden of the disease. This knowledge is particularly timely in light of rapid advancements in treatments [27], which will be crucial for advocating for new therapies and evaluating their long‐term impact on patients.

Our study aimed to describe the hidden and indirect costs faced by individuals living with NMDs and their caregivers in Canada. We sought to capture not only the nature and extent of these financial burdens but also their broader personal and social consequences, such as difficult trade‐offs and impacts on mental and physical well‐being.

## 2 Methods

### 2.1 Study Design and Rationale

This qualitative descriptive study explored the lived experiences of financial burden among individuals with NMDs and their caregivers in Canada. The study design was informed by an interpretivist paradigm and aimed to elicit in‐depth, contextualised narratives related to direct, indirect, opportunity and psychosocial costs of living with NMDs. Reporting followed the Consolidated criteria for reporting qualitative research (COREQ) checklist.

A *roundtable‐style focus group* format, defined as a semi‐structured group discussion in which participants are explicitly encouraged to respond to, question and build on one another's comments, with the moderator acting primarily as a facilitator of dialogue rather than leading a strict question–answer sequence was chosen to foster more collaborative exchange between participants, while retaining the core features of a focus group (moderated discussion based on a predefined guide).

Four virtual focus group discussions were conducted between September 2024 and April 2025, each lasting approximately 60 min and held via a secure videoconferencing platform. Three sessions included a mix of individuals and caregivers, and one session was conducted exclusively with caregivers. The rationale for this composition was twofold: mixed groups allowed us to observe how patients and caregivers jointly describe costs, and the caregiver‐only group created space for caregivers to speak about costs, trade‐offs and emotional impacts that they might feel less comfortable discussing in front of the person they support. We anticipated that these different dynamics would enrich the data by revealing both shared and role‐specific experiences. Participants were informed of the group composition in advance and provided informed consent.

### 2.2 Participants

Adults aged 18 years or older were invited to participate in focus group discussions via purposive sampling through email and social media communications through MDC (a national NMD patient organisation), which included a registration form to express interest in participating. A confirmed clinical or genetic/molecular diagnosis of any NMD or caregivers (including family members such as parents and siblings) of children with a confirmed NMD diagnosis living in Canada and completion of the Burden of Illness in NMD (BIND) study [28] were required to be eligible to participate in this study. All diagnoses were verified through MDC database that included registration forms signed by regulated health professionals, ensuring the accuracy and reliability of the participants' medical backgrounds.

### 2.3 Data Collection

The study was informed by the BIND study, a national survey of 1056 individuals living with NMDs and caregivers, including 400 participants who completed the survey by phone. While the BIND survey produced quantitative cost estimates, many respondents shared unsolicited qualitative reflections during telephone administration, describing hidden costs, system navigation challenges and psychosocial strain.

To guide the focus group discussions, we developed open‐ended, semi‐structured questions through an iterative and collaborative process. First, qualitative comments and testimonials recorded in BIND call logs were inductively coded to identify recurrent areas of financial concern, including system navigation, unmet needs for funding equipment and accommodations, and the emotional impact of economic stress. These inductive codes informed the first draft of the discussion guide, which was developed by the research leads to elicit in‐depth, experience‐based responses.

The call‐log testimonials and draft guide were then shared with three trained patient and family research partners (two adults living with NMDs and one caregiver to a young adult man with DMD). All partners had completed the imPORTND Patient‐Oriented Research Training (https://importnd.neuromuscularnetwork.ca/), a national online course developed by the Neuromuscular Disease Network for Canada and MDC. Their feedback led to refinement of question wording and sequencing to ensure relevance, accessibility and emotional safety.

The final guide focused on four core areas aligned with the study's conceptual framework: (1) types of costs not typically acknowledged or reimbursed; (2) major out‐of‐pocket expenses and challenges in obtaining funding or coverage; (3) the impact of financial challenges on daily life, employment, health and relationships; and (4) fears and concerns about current and future costs. Moderators used the guide flexibly, encouraging participants to raise additional issues and to respond directly to each other's comments.

### 2.4 Data Analysis

The focus group discussions were audio‐recorded and transcribed verbatim using a transcription service and checked for accuracy. The qualitative analysis was grounded in the cost elements of socio‐economic burden in a rare disease expert‐informed framework [27]. Transcripts were analysed using reflexive thematic analysis with an interpretivist paradigm [29]. The authors (H.O. and Z.A.) independently conducted inductive coding, after which codes were compared for consistency and refined to resolve any discrepancies. Discrepancies were discussed and resolved through consensus. Through iterative categorisation, four overarching themes and multiple sub‐themes were identified.

**Figure 1:**
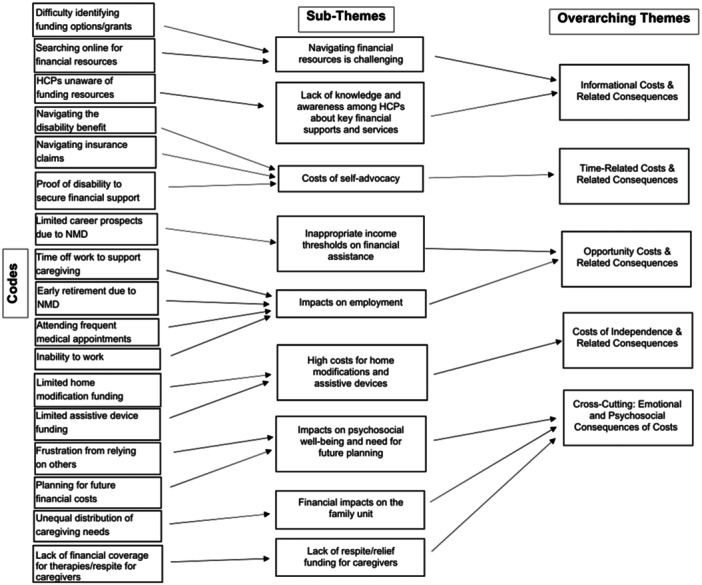
Thematic coding tree.

To enhance analytic rigour, the following steps were followed: Codebook was refined iteratively following each focus group throughout the data collection process; Saturation was assessed and deemed achieved when no new themes emerged in the final discussion; and Member checking was conducted with patient partners who reviewed theme summaries. Through an iterative process, themes were identified; sub‐themes were then developed to ensure that all statements and discussion points were systematically categorised within the overarching themes (Figure 1). Both primary analysts (PhD and MSc) are female health researchers affiliated with MDC and have formal training in qualitative methods and focus group facilitation. Their positionality as researchers within a patient advocacy organisation may have facilitated trust and openness among participants, but it also carries a risk of confirmation bias. Reflexivity was supported through analytic memo‐writing and team discussions. An external qualitative adviser, not employed by MDC and with experience in health services research, reviewed the theme structure and definitions to minimise interpretive bias.

## 3 Results

### 3.1 Participant Demographics

A total of 58 individuals participated in four virtual focus group roundtable discussions. Participants ranged in age from 23 to 67 years (mean = 41). Of these, 76% (*n* = 44) were individuals living with NMDs and 24% (*n* = 14) were caregivers of children or adults with NMDs. Three sessions included both patients and caregivers, while one session was conducted exclusively with caregivers to allow for open discussion of caregiver‐specific financial and emotional burdens. Caregivers included parents, spouses/partners, siblings and extended family members.

Most participants self‐identified as White (approximately 90%), with smaller proportions identifying as East Asian or South Asian; fewer than 1% identified as Black, Indigenous or another racialised group. Participants resided across Canada, with 62% living in urban areas and 38% in rural or remote communities. A broad range of neuromuscular conditions was represented, including DMD (10), MG (4), SMA (6), facioscapulohumeral muscular dystrophy (5), limb‐girdle muscular dystrophy (6), Charcot–Marie–Tooth disease (3), Friedreich ataxia (3), centronuclear myopathy (2), myofibrillar myopathy (2), oculopharyngeal muscular dystrophy (6), congenital muscular dystrophy (3), myotonic dystrophy type 1 (5), inclusion body myositis (2) and nemaline myopathy (1). Educational attainment was high: all participants had completed at least high school, most had post‐secondary education, and 14% held a graduate or professional degree.

Among participants with SMA, 67% (4 of 6) were receiving or had previously received a disease‐modifying therapy (nusinersen, onasemnogene abeparvovec or risdiplam). Household income data were collected in the larger quantitative BIND survey, but were not consistently available for all qualitative participants; income bands are therefore not reported for this subsample.

At the time of participation, none of the individuals or children represented by caregivers required invasive or non‐invasive ventilation, enteral feeding, or had a diagnosed cognitive impairment or learning disability. These factors are known to substantially increase care complexity and cost and may therefore influence the financial burden experienced.

### 3.2 Thematic Analysis

**Figure 2:**
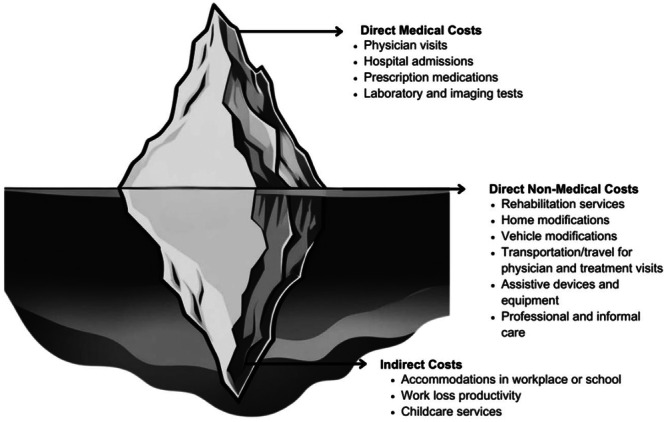
Direct medical, direct non‐medical and indirect costs associated with NMDs.

The experiences of living with and/or caring for a person with an NMD were categorised into time‐related, informational, independence and opportunity costs. Importantly, the costs identified were often direct non‐medical or indirect in nature (Figure 2).

#### 3.2.1 Informational Costs and Related Consequences

##### 3.2.1.1 Navigating Financial Resources Is Challenging

Participants identified that the landscape of financial resources, including information on eligibility criteria for critical funding programmes for persons with disabilities for equipment and rehabilitation services, is highly complex and challenging to navigate. Most participants reported significant effort and frustration in identifying the most suitable funding programme for themselves or their children/family members, finding that the available programmes were often confusing and inaccessible. When financial assistance was available, it often covered only part of the costs for essential equipment, requiring combining multiple funding sources and additional out‐of‐pocket expenses. Participants also stressed the importance of carefully managing their expenses, invoices and payments and following funding rules. Keeping accurate records and understanding the nuances of different funding rules were crucial for avoiding delays, ensuring proper reimbursement and maximising available support. This ongoing effort added to the overall administrative burden and complexity of securing and managing financial assistance.

Since my son's diagnosis, finances have become strained, and I've taken on the role of the family “Project Manager.” I use an Excel spreadsheet to track all expenses related to his condition and still find myself spending extra on accounting and legal services to ensure our tax returns are correct and acceptable.

Parent of a child with SMA

##### 3.2.1.2 Lack of Knowledge and Awareness Among HCPs About Key Financial Supports and Services

HCPs, including neurologists, physiatrists, family physicians, physiotherapists (PTs), and occupational therapists (OTs), were often viewed as gatekeepers to essential funding resources. Participants reported a prevalent perception that these professionals frequently lack knowledge and awareness about available funding and disability supports, which complicates access to these resources for individuals and their families. Many participants found that their healthcare providers were unfamiliar with the funding options (such as government programmes, non‐government funding agencies within provinces, and patient organisations like MDC) and unable to offer appropriate referrals. Additionally, the insufficient familiarity with disability funding supports and services by health professionals often led to delays in completing necessary forms for access to funding or disability related benefits and meant that participants were paying out of pocket to access services such as private doctors or tax specialists in some cases to aid in completing necessary documentation. This process involved scheduling appointments, waiting for evaluations or reports, and dealing with potential delays or follow‐ups, which added to the overall burden of managing a disability and contributed to the indirect costs associated with securing support and funding.

Investing in a specialist to complete our son's disability tax credit forms was well worth the expense, rather than relying on our family doctor. The additional cost was essential to ensure that everything was completed accurately.

Parent of a child with DMD

It often seems like you need to provide an overwhelming amount of documentation and evidence just to show that you have a neuromuscular disorder and you need help. The bureaucratic hurdles make it feel as though you're fighting an uphill battle to get the financial support you need.

Person with Facioscapulohumeral muscular dystrophy

#### 3.2.2 Time‐Related Costs and Related Consequences

##### 3.2.2.1 Costs of Self‐Advocacy

Participants noted that while costs are often quantified in terms of expenses for assistive devices, home modifications or supportive treatments, there are additional costs related to the time spent advocating for resources and navigating funding programmes. Navigating financial aid, securing necessary signatures and self‐advocating require substantial time, energy and resources—demands that often take a backseat to immediate caregiving needs. The requirement for confirmation of disability, which requires a signature from a regulated health professional for various funding applications, was identified as a significant time‐related cost. As a result, many are forced to choose between paying out‐of‐pocket or going without critical supports due to prohibitive costs. Additionally, the burden of caregiving and the necessity of maintaining employment for financial stability frequently limit the ability to seek financial assistance or advocate for financial aid.

I think it's fair to say that that I turn over every rock to try to find stuff and it's unique to me. I understand that most people can't do that, and if you can't do that, you're going to really end up with a lot of costs. I also will fight and fight and fight to get the funding so it doesn't come out of pocket.

Person with Charcot‐Marie tooth disease

It took me 8 months to get the OT to come by for a chair, for the shower, and by the time they showed up with the chair, it was useless because he had progressed. And the things that I really need, well, that's not covered.

Parent of a child with Friedreich ataxia

#### 3.2.3 Opportunity Costs and Related Consequences

##### 3.2.3.1 Inappropriate Income Thresholds on Financial Assistance

A major barrier faced by people with NMDs is the stringent income thresholds set by financial support programmes. Despite the availability of these programmes, many find themselves ineligible due to even modest family incomes. Participants noted that low‐income thresholds limited access to government funding for services or assistive devices/technology, thus resulting in significant income loss. Some participants shared that increasing their income and assets would mean that their financial support for equipment or home modifications would be diminished. This situation forces many individuals with NMDs to bear large out‐of‐pocket expenses for essential services and assistive devices, regardless of their income level, and limits their ability to advance their careers without losing financial support.

I found like a lot of programs aimed to support disabled people in the community require you to have a very, very low family income. My husband doesn't make a ton of money, he works for a grocery store, but he makes enough money that that means I wouldn't qualify for social assistance.

Person with MG

I am proud of having worked for as long as I could especially given my circumstances. However, the fact that I worked and had assets, disqualified me from the Ontario Disability Support Program which would cover a lot (perhaps all) of the disability‐related expenses and for which I now have to pay myself.

Person with SMA

##### 3.2.3.2 Impacts on Employment

For many participants, maintaining employment posed significant challenges due to the need for workplace adaptations, flexible working conditions and understanding employers. As their NMDs progressed, some were forced to leave the workforce, resulting in the loss of crucial employer‐sponsored healthcare benefits. Others reported needing to take early retirement, as the fatigue resulting from their condition increasingly limited their ability to meet job requirements. The transition out of the workforce often left individuals without adequate financial support during retirement, pushing many towards or below the poverty line despite their efforts to remain self‐sufficient.

As a nurse, I had to retire early because I no longer had the strength to manage even the adjusted duties. This was unexpected and really hit hard on our finances and savings plan.

Person with Oculopharyngeal muscular dystrophy

Caregivers reported substantial opportunity costs, either having to leave their jobs or declining employment opportunities due to their caregiving responsibilities and lack of flexibility from employers. This often left their families relying on a single income, further intensifying their financial strain. Those who managed to keep their jobs described the financial impact of lost income from taking time off or adjusting their work schedules to meet their family members' needs. The time spent accessing services, coordinating care and managing hospital visits and appointments was likened to a full‐time job, making it unsustainable to maintain both work and caregiving responsibilities.

Basically, I'm not really working. I'm managing the household, all of the school issues, the hospital visit issues, the physio issues. We don't have enough time in the day. You know, as a full‐time worker, you wouldn't be able to do it.

Parent of a child with Friedreich ataxia

I had to quit my full‐time job to become my son's primary caretaker and do homeschooling since he was getting sick all the time. This was a loss of income*.*

Parent of a child with Congenital Muscular Dystrophy

Some participants shared it was imperative to remain in the workforce to afford essential supports and services. Many described staying in full‐time jobs or delaying retirement despite the progression of their NMD, as their income was vital not only for covering the costs of care, equipment and services but also for meeting basic needs such as groceries and home maintenance.

In addition to significant expenditures for home modifications and assistive devices, participants highlighted inadequate financial assistance for accessing critical rehabilitation services such as PT, OT, massage therapy and so forth. Even those with employment benefits faced substantial out‐of‐pocket expenses for long‐term access to rehabilitation services, as their insurance coverage typically extended to only a limited number of sessions.

For many, maintaining employment was driven by the need to retain healthcare benefits, which partially offset the costs of treatments, assistive devices and medications—expenses that would otherwise be unaffordable. However, for others relying on private insurance, these essential services were often excluded from coverage, leading to increasing financial burdens over time in addition to the premiums already paid for insurance.

My main concern is primarily the expense of physiotherapy which is a necessity and unfortunately, insurance companies generally cover only 300 dollars a year and one session alone is typically 120 dollars for 1 h and I need two sessions a week at a minimum to maintain my muscle strength.

Person with Limb‐girdle muscular dystrophy

I'm looking for a new job with good benefits or better benefits than I have now, but for now I'm stuck in a job I don't really like, but it has decent benefits.

Person with Charcot‐Marie tooth disease

I am not able to retire because my adult daughter receives life‐changing treatment through my health insurance plan.

Parent of an adult with SMA

#### 3.2.4 Costs to Independence and Related Consequences

##### 3.2.4.1 High Costs for Home Modifications and Assistive Devices

A major financial burden for participants with NMDs involved modifying their homes to meet evolving accessibility needs and purchasing assistive devices needed to support their independence. For many participants, these costs were unexpected, and they did not realise they would need to cover them entirely out of pocket. The progressive nature of their condition often necessitated frequent purchases of new equipment or further home modifications, which were frequently not aligned with the funding timelines of agencies and government programmes. Additionally, when participants did access funding programmes, they often faced long waits for funding approval, forcing them to consider paying out of pocket. Some participants described large expenses for specific pieces of equipment, which amounted to tens of thousands of dollars.

Vehicle and home modifications funding are only applicable to low‐income families, not based on the needs of the child. All home and vehicle modifications are out of pocket for us. This means our kids are not getting all the right tools for success since we can only pay what we can afford as needed.

Parent of a child with congenital muscular dystrophy

The discussions revealed that built environments do not accommodate those with mobility limitations, placing the financial burden of necessary modifications on the individuals themselves. Participants noted that modifying rental homes or apartments was nearly impossible, as landlords typically refused to make changes or to provide financial support. Consequently, participants often had to endure living in inaccessible homes and struggled to find affordable, accessible housing. They reported that the high costs of home modifications frequently left them in unsafe living conditions, which negatively impacted their well‐being and led to additional expenses from potential injuries. Additionally, the high anticipated costs led them to forego necessary home modifications, despite their importance for safety and well‐being, due to concerns about affordability.

My bathroom is the size of most people's linen closets. And the quote is $32,000 for something that is probably no more than maybe 8 feet by 4 feet. I cannot use my bathroom. In order to do so, I have to use a mechanic stool. To transfer from my chair to the mechanic stool to get through the bathroom door, I had an accident 3 weeks ago and ended up an emergency room

Person with Charcot‐Marie tooth disease

Participants stressed that independence was a top priority, but it often involved significant costs, especially as their NMDs progressed. They detailed how their income was directed towards accessing services and supports necessary for activities such as entering their homes, shopping for groceries or driving their vehicles, as well as activities of daily living. For example, some participants mentioned spending on premium taxi services to navigate their communities independently.

"It's incredibly frustrating. While others can save their money for enjoyment, I find myself spending more just to be able to step outside my front door. I had to buy a larger vehicle, and people often make sarcastic comments about me getting an SUV, as if it's a luxury. But for me, it's about accessibility and independence … being able to get in and out of a car on my own."

Person with Limb‐girdle muscular dystrophy

Parents of children with NMDs expressed concerns about their children's opportunities for success in both school and the workplace, particularly for those with NMDs associated with cognitive impairments or an elevated risk of intellectual or learning disability. They were often advised to enrol their children in specialised schools for disabilities, but they preferred mainstream education to ensure their children could interact with their peers. This choice, however, came with extra costs to support their children's specific needs. While parents hoped for their children to achieve independence from a young age through adulthood, they had to carefully consider the level of support their child would require away from home and the associated financial implications.

#### 3.2.5 Cross‐Cutting

Emotional consequences and mental strain cut across all themes.

##### 3.2.5.1 Impacts on Psychosocial Well‐Being and Need for Future Planning

Participants highlighted the emotional and social strain caused by living with or caring for someone with an NMD. They described how the overwhelming financial burden of managing their condition significantly affected their mental well‐being, with caregivers expressing an even greater impact. Despite their desire to seek mental health support, such as counselling from psychologists or psychiatrists, many found these services unaffordable due to insufficient funding, further compounding their stress.

Access to mental health for treating my depression relating to my NMD ended when the Province of Nova Scotia stopped funding this type of mental health support.

Person with Myotonic Dystrophy

Participants reported frequently having to manage both their current financial responsibilities and future financial planning, which further exacerbated the impacts on their mental health. They expressed concerns about anticipated high costs related to home modifications and assistive devices. Many participants mentioned making financial decisions based on the expected progression of their NMDs, such as staying in full‐time employment to accumulate a sufficient retirement fund or seeking jobs with better retirement benefits to ensure they could cover future expenses, including home adaptations and assistive devices.

My husband and I don't have pensions. I am working still, because we are trying to continue to save because I know I'm going to have so many expenses in the future.

Person with Inclusion body myositis

Participants who were caregivers expressed concerns about whether their family members would be able to be financially independent in the future as their NMDs progress over time. Parents of young adults with NMDs shared that seeking an education that will allow their children to participate in the workplace and earn an income in order to afford the supports they require when they get older posed a significant challenge:

I worry about her because she's not able to work. I look at her trying to find a job is very, very challenging because people see her as lazy or not trying hard enough. Seeing my young adults right now, that is their big biggest barrier, is getting a good education in order to get into the workplace.

Parent of a child with Myotonic dystrophy

**Figure 3:**
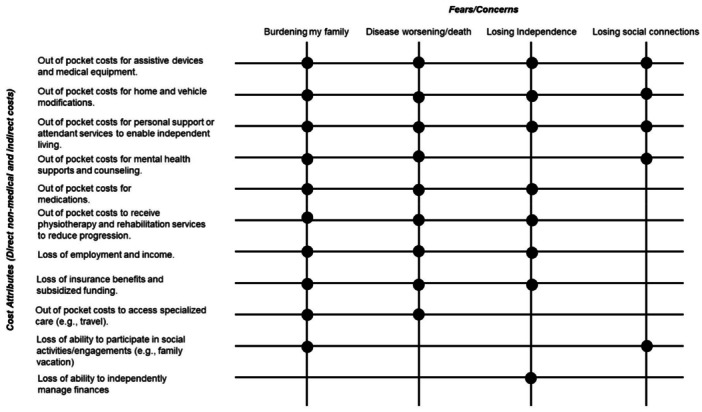
Patterns in fears associated with the financial burden of NMDs.

Across discussions, four recurring fears emerged: becoming a burden on one's family, disease progression leading to early death, loss of independence and social isolation (Figure 3). These fears were closely tied to the financial strain resulting from the various costs associated with their condition. Financial insecurity heightened participants' emotional distress, eroded their sense of control and limited their capacity for future planning or engaging fully in daily life. By explicitly connecting these cost‐related challenges to the broader psychosocial fears, the findings underscore the profound impact of financial burdens on the lived experiences of individuals with NMDs. This psychological impact was reported regardless of income level or disease type, underscoring the pervasive and hidden nature of the burden.

##### 3.2.5.2 Financial Impacts on the Family Unit

Most participants reported that financial stressors profoundly affected their family dynamics and relationships, particularly regarding the costs and logistics of care. These financial pressures often created divisions within the family unit. While peers could allocate income to leisure activities like vacations, participants frequently felt constrained by the need to prioritise their financial obligations. Parents described the constant challenge of balancing resources to support both children with NMDs and those without, striving to ensure that siblings without NMDs did not miss out on opportunities. Conversely, participants with siblings who had NMDs often recounted having to forego extracurricular activities or family vacations as resources were primarily directed towards the sibling with NMD.

There were lots of things that we couldn't afford. I didn't grow up going to any extracurricular as my dad was a sole provider and my mom became a full‐time caregiver. She couldn't work. So, we really relied on my dad.

Sibling of a person with DMD

Caregivers emphasised that the financial burden extended beyond supporting individuals with NMDs to include their own well‐being. Many described the high costs of accessing essential treatments, such as physiotherapy, massage therapy or chiropractic care, which were necessary to manage the physical demands of caregiving. However, these services were often financially out of reach. Caregivers also highlighted the additional strain of leaving full‐time employment to assume caregiving responsibilities, resulting in the loss of employment benefits that previously covered vital services like physiotherapy or psychotherapy. This shift frequently forced them to pay out of pocket for their care, further exacerbating financial instability and negatively impacting their health. Participants expressed an urgent need for targeted financial assistance to ensure caregivers could access these necessary supports while maintaining their ability to care for their family members.

##### 3.2.5.3 Lack of Respite/Relief Funding for Caregivers

Caregivers in this study noted a significant shortfall in funding for respite or relief care. When such funding was available, it often proved inadequate for sustaining families over an extended period. Participants reported that the demands of caregiving became increasingly strenuous as their child's NMD progressed, and they expressed concern over the insufficient financial support for respite services. Without adequate funding to access relief, caregivers emphasised that they would struggle to maintain their ability to support their family members effectively.

I'm burned out and exhausted. So, if there's no respite care and if I get run down and sick, I have my own immune issues. How am I going to be able to continue helping them if I know have no help?

Caregiver of family members with Charcot‐Marie tooth disease

## 4 Discussion

This study highlights that the most burdensome costs experienced by individuals with NMDs and their caregivers remain invisible to healthcare systems and payers. Participants described financial strain that extended well beyond out‐of‐pocket expenses, encompassing administrative burden, time loss and psychosocial impacts that affected physical health, mental well‐being, social participation and long‐term opportunities. These findings highlight how structural gaps in Canada's mixed public–private system shift substantial financial and emotional responsibility onto families.

Although Canada's publicly funded system covers hospital care, physician services and diagnostic testing, it provides limited coverage for mobility equipment, respiratory devices, home modifications and community rehabilitation—the very supports that individuals with progressive NMDs rely on to maintain function and independence. Consistent with our findings, participants described substantial out‐of‐pocket expenditures and significant time navigating fragmented provincial funding programmes. These experiences mirror broader national patterns: assistive devices are inconsistently funded across provinces, reimbursement criteria are complex, and many families are left to self‐fund essential equipment or seek partial co‐payment assistance from donor‐supported patient organisations.

As NMDs progress, patients and their caregivers are faced with newer and more difficult challenges to navigate financially, including loss of ambulation resulting in the use of assistive devices or changes in bulbar and respiratory function that impact breathing, speech and swallowing, thus necessitating adaptations to suit these new needs, which can be frequent and unpredictable. As more people with NMDs are living longer in their homes rather than in institutional settings, caregiving demands intensify. These responsibilities are overwhelmingly shouldered by informal caregivers, yet 57% of COI studies in rare diseases do not account for informal care costs [30]. Caregivers in our study described reduced employment, foregone career advancement and substantial emotional burden. These findings align with prior research showing that informal care is a major contributor to the overall financial burden [31]. For example, studies in DMD report that many caregivers leave the workforce entirely, with one study finding that 29% of parents stopped working and 38% reduced their working hours to provide care [32, 33]. Importantly, some caregivers themselves were living with NMDs, compounding both the emotional and financial strain. These findings underscore the need for caregiver‐specific supports, including respite, financial subsidies and workplace accommodations.

Governmental and insurance financial supports and services have not kept pace with the rising costs of care, forcing individuals with NMDs and their families to absorb the financial burden of funding pharmacological treatments and essential services that support health, daily activities and independent living. In fact, CIHI has reported that healthcare spending in Canada is expected to exceed economic growth, and currently, the average per‐person spending on healthcare is among the highest internationally [8]. In the absence of cures, and with the high cost of disease‐modifying drugs for some NMDs, many individuals with NMDs rely on rehabilitation services, such as medical equipment and physiotherapy, to maintain their well‐being [34]. These services are typically not funded by the public healthcare programmes in Canada, resulting in most individuals paying out of pocket [7]. Importantly, assistive devices and rehabilitation services remain unaffordable, leaving many to either go without necessary care or spend considerable time navigating the healthcare system in search of financial support. As a result, cost‐related non‐adherence to treatment becomes a significant risk, leading to worsened disease progression, increased hospitalisations and a diminished quality of life. While research specifically focusing on cost‐related non‐adherence to assistive devices and rehabilitation therapies is limited, this study indicates the same principles likely apply: the unaffordability of equipment and rehabilitation services can lead individuals to forgo or delay their use, ultimately worsening health outcomes and increasing long‐term healthcare costs.

This study has a few limitations: Purposive sampling was used to recruit participants with diverse diagnoses and backgrounds; individuals were drawn from a single national organisation (MDC), potentially introducing selection bias. Participants may have been more resource‐aware or engaged than the broader NMD population. While the study captured a range of NMDs and disease severities, it did not disaggregate themes by disease stage or phenotype, which may influence financial burden. This heterogeneity reflects the diversity of NMDs but also limits the ability to generalise findings across specific subgroups. While qualitative research is not intended to be generalisable in the statistical sense, the insights captured here may not reflect all financial experiences. Data saturation was considered reached when no new themes emerged in the final focus group, but we acknowledge that additional discussions might have revealed further nuances. Finally, although participants reported on sibling and family‐level impacts, many perspectives represented in the analysis were those of patients and parents; future research could explore the perspectives of other family members more systematically. Furthermore, participant recruitment through MDC's database was also limited by a lack of interest [35, 36, 37, 38]. For instance, the perception that participation in the study would not confer any further benefits directly to the participant, as well as, lack of health literacy, which leads to a lack of understanding of the research purpose, and a lack of communication between the researcher and participants, particularly for those registered without any specific contact information.

This study offers a unique, patient‐driven perspective on the real‐life socio‐economic burden associated with NMDs. A key strength of the study lies in the active engagement of many individuals with NMDs and their caregivers, motivated by a desire to improve outcomes for both themselves and the broader community. The trust established through MDC, a patient advocacy organisation, was crucial in facilitating nationwide data collection, ensuring that insights were both diverse and representative. The study's design also integrated wrap‐around supports, ensuring that participants not only shared their experiences but were also provided with practical resources. For example, individuals unable to afford essential equipment, such as wheelchairs, were connected to MDC's Equipment Programme to offset expenses. This approach highlights the importance of partnering with patient organisations, particularly for studies involving rare diseases, to address sensitive financial discussions in a trusted and supportive environment while simultaneously offering tangible support.

These findings support several actionable strategies. Clinically, providers should proactively address the administrative, emotional and financial burden of navigating equipment, funding and home‐care supports. Embedding care coordinators or social workers into NMD clinics may alleviate some of these hidden costs. At the policy level, more inclusive and streamlined provincial assistive‐device programmes are needed, along with expanded eligibility for federal supports such as the Disability Tax Credit. Simplifying application processes, increasing funding for home and mobility equipment, and boosting respite and mental health support for caregivers could substantially reduce the cumulative burden identified in this study [39, 40]. Broader adoption of national rare‐disease frameworks that emphasise navigation support, equitable access and reduction of administrative burden would further strengthen care for individuals with NMDs and their families.

## 5 Conclusion

This study highlights the significant but often under‐recognised out‐of‐pocket expenses faced by individuals with NMDs and their families, including increasing reliance on assistive devices and escalating caregiving demands as conditions progress. These pressures compound financial, emotional and social stressors. Hidden costs such as informational burdens, administrative navigation, lost employment opportunities and threats to independence were central to participants' lived experience of financial strain. These findings underscore the need for policies that expand equitable access to financial supports, rehabilitation services and assistive technologies and reaffirm the importance of a healthcare system responsive to the economic realities of people living with NMDs.

## Author Contributions

**Homira Osman:** conceptualization, methodology, formal analysis, investigation, writing – original draft, writing – review and editing, visualization. **Zainab Adamji:** conceptualization, methodology, formal analysis, investigation, writing – original draft, writing – review and editing, visualization. **Stacey Lintern:** conceptualization, methodology, formal analysis, investigation, writing – original draft, writing – review and editing, visualization. **Ian C. Smith:** conceptualization, methodology, formal analysis, investigation, writing – original draft, writing – review and editing, visualization. **Alyssa Grant:** conceptualization, methodology, formal analysis, investigation, writing – original draft, writing – review and editing, visualization. **Lola E.R. Lessard:** conceptualization, methodology, formal analysis, investigation, writing – original draft, writing – review and editing, visualization. **Hanns Lochmuller:** writing – review and editing. **Hugh McMillan:** writing – review and editing. **Kathryn Selby:** writing – review and editing. **Gerald Pfeffer:** writing – review and editing. **Lawrence Korngut:** writing – review and editing. **Cynthia Gagnon:** writing – review and editing. **Kednapa Thavorn:** conceptualization, methodology, formal analysis, investigation, writing – original draft, writing – review and editing, visualization. **Jodi Warman‐Chardon:** conceptualization, methodology, formal analysis, investigation, writing – original draft, writing – review and editing, visualization.

## Ethics Statement

The study protocol was approved by the Ottawa Health Science Network Research Ethics Board (Protocol ID #20210601‐01H), and research was conducted in accordance with the ethical standards as laid down in the 1964 Declaration of Helsinki.

## Consent

All quotes are included with the expressed consent of the participant. All participants provided verbal consent prior to participating in the focus group discussions.

## Conflicts of Interest

The authors declare no conflicts of interest.

## Data Availability

The data that support the findings of this study are available on request from the corresponding author. The data are not publicly available due to privacy and ethical restrictions.
